# Using functional genomics to advance the understanding of psoriatic arthritis

**DOI:** 10.1093/rheumatology/keaa283

**Published:** 2020-08-10

**Authors:** Chenfu Shi, Magnus Rattray, Anne Barton, John Bowes, Gisela Orozco

**Affiliations:** k1 Division of Musculoskeletal and Dermatological Sciences, Faculty of Biology, Medicine and Health, Centre for Genetics and Genomics Versus Arthritis; k2 Division of Informatics, Imaging and Data Sciences, Faculty of Biology, Medicine and Health, University of Manchester; k3 NIHR Manchester Biomedical Research Centre, Manchester University NHS Foundation Trust, Manchester Academic Health Science Centre; k4 Lydia Becker Institute of Immunology and Inflammation, Faculty of Biology, Medicine and Health, University of Manchester, Manchester, UK

**Keywords:** psoriatic arthritis, genetic susceptibility, GWAS, functional genomics, causal genes, regulation of gene expression, chromatin conformation

## Abstract

Psoriatic arthritis (PsA) is a complex disease where susceptibility is determined by genetic and environmental risk factors. Clinically, PsA involves inflammation of the joints and the skin, and, if left untreated, results in irreversible joint damage. There is currently no cure and the few treatments available to alleviate symptoms do not work in all patients. Over the past decade, genome-wide association studies (GWAS) have uncovered a large number of disease-associated loci but translating these findings into functional mechanisms and novel targets for therapeutic use is not straightforward. Most variants have been predicted to affect primarily long-range regulatory regions such as enhancers. There is now compelling evidence to support the use of chromatin conformation analysis methods to discover novel genes that can be affected by disease-associated variants. Here, we will review the studies published in the field that have given us a novel understanding of gene regulation in the context of functional genomics and how this relates to the study of PsA and its underlying disease mechanism.

**Rheumatology key messages**

- It is challenging to translate GWAS results into patient benefit.
- Functional genomics can elucidate the role of GWAS variants in disease.
- Understanding the function of GWAS variants can lead to the discovery of new treatments.

## Introduction

Psoriatic arthritis (PsA) is a chronic autoimmune disease with a high disease burden [1–4] and major impacts both to the patients’ quality of life and economic and social impact to the society. It is characterized by a combination of psoriasis and arthritis. The disease is clinically heterogeneous, causing it to be frequently confused with similar diseases such as other forms of arthritis or psoriasis [5, 6]. The biological mechanism behind the disease is not well understood, but it is known that PsA has a strong, complex genetic component with a very high genetic heritability [7, 8]. Family studies have shown that people with a first degree relative affected by PsA have 30–55 times the risk of developing PsA compared with the general population [9–13].

Genome wide association studies (GWAS) have identified a significant part of the genetic factors that lead to the disease [14–19] (Table 1). The main association is with HLA class I genes, which was already discovered by earlier family studies [21]. A meta-analysis published in 2015 subsequently identified the loci that distinguish PsA from cutaneous psoriasis [14]. For example, PsA lacked associations with *TNFRSF9* and *LCE3C*/*B* that are present in psoriasis and has different HLA-C associations. Importantly they found that, in the *IL23R* and *TNFAIP3* loci, PsA had independent signals compared with psoriasis, suggesting a different mechanism acting in the same loci. Nevertheless, the majority of the loci still overlap with psoriasis. In the same year, a larger study with almost 2000 PsA patients was published [19]. In this study they used the custom genotyping chip Immunochip, which targeted specific autoimmune-associated loci with a much higher resolution and identified many genome-wide significant loci, such as the 5q31 loci that is specific to PsA (Table 1).

**Table 1:** Currently known genome-wide significant (*P*-value < 5 × 10^−8^) GWAS loci for PsA outside the HLA locus [14, 19]

Loci	Lead SNP	Putative candidate gene
chr1: 24192153 [14]	rs7552167	*IFNLR1*
chr1: 67135003-67193271 [19]	rs12044149	*IL23R*
chr1: 113834946 [20]	rs2476601	*PTPN22*
chr5: 132083255-132220510 [19]	rs715285	*P4HA2*
chr5: 151085340-151093041 [19]	rs76956521	*TNIP1*
chr5: 159337169-159339014 [19]	rs4921482	*IL12B*
chr6: 31317371 [14]	rs12191877	*RPL3P2, WASF5P*
chr6: 111259358-111587679 [19]	rs33980500	*TRAF3IP2*
chr12: 56116134-56360038 [19]	rs2020854	*STAT2, IL23A*
chr14: 34756277-35418710 [14]	rs8016947	*NFKBIA*
chr19: 10349293-10366391 [19]	rs34725611	*TYK2*

Multiple associations are present in HLA-A, B and C, which are also the strongest associations. The putative candidate genes were mostly determined by mapping the closest or overlapping gene, which might be inaccurate. Coordinates in hg38 genome build. Many loci such as rs9321623 (*TNFAIP3*) had a *P*-value of 6 × 10^−8^ so they have been omitted from this table.

## Linking variants to function

Despite the success of GWAS studies in identifying the genetic variants that are linked to PsA, understanding how the associated genetic variants affect the underlying biological mechanisms is not straightforward. Only a small proportion of the variants associated with complex traits identified by GWAS affect coding sequence of proteins. Farh *et al.* [22] produced an important study in this field, mapping GWAS signals from 21 autoimmune diseases and estimating that 90% of them affected non-coding regulatory regions with the majority (60%) affecting enhancer regions in immune related cells [22]. This makes understanding the disruptive effect of disease-associated variants intricate because many of these regulatory elements can act at long range through chromatin interaction mechanisms [23–30]. Other mechanisms are also possible in a minority of the loci, such as variants affecting long non-coding RNAs (lncRNA) and microRNAs (miRNA). For example, in rheumatoid arthritis there have been reports of genetic variants affecting the miRNA miR-146a [31] and the lncRNA C5T1lncRNA [32].

**Fig. 1:**
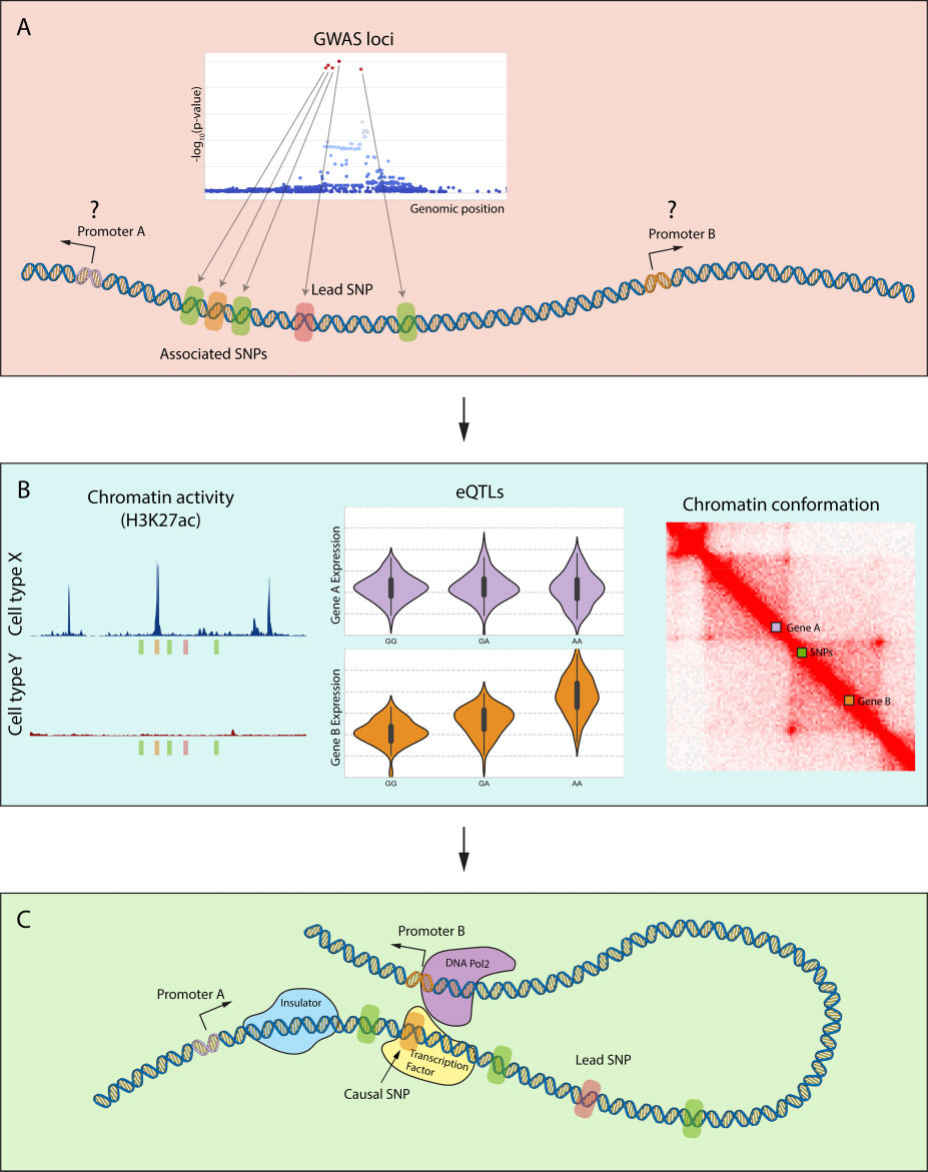
Using functional genomics to describe GWAS loci (**A**) A typical GWAS loci usually consists of many variants in high linkage disequilibrium and frequently far away from any genes, which can make the interpretation of the association challenging. (**B**) It is possible to use a combination of functional genomics techniques to study these loci, such as: chromatin activity to identify which SNPs are functionally relevant and in which cell types; eQTLs to correlate genotype with changes in gene expression; and chromatin conformation to identify regulatory domains that determine which genes can be affected. (**C**) These methods combined with others allow us to identify the functional importance of GWAS associations in the disease.

The simplest method to link variants to functional effect and their target genes is correlating the genetic makeup of different individuals with the expression levels of genes in a specific tissue or cell type (expression quantitative trait loci or eQTL) (Fig. 1B). The most comprehensive study in this regard is the Genotype-Tissue Expression (GTEx) project, which analysed a large number of tissues from recently deceased people and correlated RNA expression levels with their genotype [33]. More specific studies with larger sample sizes were done with whole blood [34–36] and other immune cells [37–39], discovering tens of thousands of genetic variants that regulate gene expression. Although there are several limitations, such as the need of large sample sizes to draw statistically significant conclusions and the high cost, eQTL studies have been fundamental in describing many principles of gene regulation, including:

- Some eQTLs are stimulation responsive and cell-type specific [38, 40–44]. For example, Schmiedel *et al.* found that 41% of the identified genes showed a strong cis-association with genotype only in a single cell type out of the 13 analysed [38], while Fairfax *et al.* showed that more than half of the eQTLs identified on primary monocytes were apparent only after stimulation [44]. This demonstrates that experiments on cells in resting state will not show all the possible associations.
- eQTLs do not always link the nearest gene to the single-nucleotide polymorphism (SNP), so it is not accurate to assign the disease-associated variants to the nearest gene [35, 42, 43].

Many studies have tried to link disease-associated variants to genes in specific cell types, especially for autoimmune diseases. Because eQTLs are context specific, some studies have used cells derived from patients to discover specific links with disease associated loci. For example, Thalayasingam *et al.* mapped eQTLs in CD4+ T cells and B cells from 344 patients with untreated RA identifying a number of candidate genes linked to variants associated with RA [45]. In PsA, Bowes *et al.* mapped their novel disease associated loci using cell-type specific eQTLs from CD4+ and CD8+ primary T cells [19]. Another study mapped eQTLs in skin tissues from psoriasis patients and looked at overlap with psoriasis GWAS results, finding significant enrichment of psoriasis GWAS SNPs in their eQTL dataset, with effects on the expression of genes such as *FUT2, RPS26* and *ERAP2* [46]. Immune cells are of a particular interest in autoimmune diseases and a large number of disease-associated variants have been found to overlap eQTLs in these cells [38, 47]. A summary of eQTLs overlapping disease associated variants in PsA is reported in Table 2.

**Table 2:** Currently known immune and skin eQTL signals overlapping PsA associated variants

Variant	Genes
rs12044149	*IL23R* [35], *MIER1* [35]
rs715285	*SLC22A5* [33, 35, 36, 38], *DC42SE2* [35], *P4HA2* [35, 39], *IRF1* [35], *AC116366*.*6* [33, 35], *SHROOM1* [35], *LYRM7* [35], *RAD50* [35], *KIF3A* [35], *PDLIM4* [33]
rs76956521	*TNIP1* [35, 39], *SLC36A1* [35]
rs4921482	*RNF145* [35]
rs33980500	*KIAA1919* [35], *C6orf3* [33, 35], *REV3L* [35], *TRAF3IP2* [39]
rs2020854	*SPRYD4* [35, 36, 39], *STAT2* [35, 36], *CNPY2* [35, 36, 39], *RBMS2* [35, 36], *PAN2* [35], *SUOX* [35], *MYL6B* [35], *COQ10A* [35], *STAT6* [35], *CDK2* [35], *RPS26* [35], *CS* [39]
rs8016947	*KIAA0391* [35, 36], *SRP54* [35], *PPP2R3C* [35, 39]
rs34725611	*TYK2* [35, 39], *ICAM3* [35], *CDC37* [35], *TMED1* [35], *SLC44A2* [35], *PDE4A* [35], *KEAP1* [35], *MRPL4* [35], *AP1M2* [35], *PDE4A* [38], *CTC-215O4.4* [38]
rs7552167	*IFNLR1* [33, 35, 38], *IL28A* [36]
rs12191877	*MICB* [36, 38], *HCG27* [38], *HLA-S* [33], *PSORS1C1* [33], *HLA-B* [33], *ZBTB12* [33], *CDSN* [33]

Data was collected from the following relevant databases: eQTLgen [35], DICE [38], GTeX v7 [33], Westra 2013 [36] and Lappalainen 2013 [39].

However, eQTL studies have failed to capture all GWAS loci and, although GWAS SNPs are significantly enriched in eQTLs [34, 38, 44, 48], only 20–50% of GWAS SNPs overlap with an eQTL. Moreover, a major drawback of eQTLs is that they only prove correlation and not causation and are also biased towards large effect sizes.

Variations of QTL analysis methods have been proposed that correlate genotype with other factors such as histone modification levels (hQTL) [49, 50] and chromatin accessibility (caQTL) [51–53], and, as expected, many eQTL were also hQTLs and caQTLs. More interestingly, Alasoo *et al.* showed that 60% of stimulus-specific eQTLs were caQTLs in naïve cells, suggesting that they could perturb enhancer priming [40].

An alternative to eQTL analysis is to functionally describe the mechanisms by which variants can affect genes; for example, using chromatin conformation techniques.

## Using chromatin conformation methods to map target genes

As stated previously, the majority of disease-associated variants are predicted to affect regulatory regions such as enhancers [22]. Enhancers are regulatory elements that are bound by transcription factors and have long been known to regulate genes by long-range effects [29, 54–56]. These elements were initially identified from viral sequences and were found to ‘enhance’ transcription of nearby elements [57]. Identifying enhancers is challenging due to the lack of accurate computational prediction methods, and due to the fact that they are very context and cell-type specific [40, 41, 58]. Identification of enhancers is frequently done by probing the presence of histone tail modifications or the presence of bound transcription factors [58, 59]. Tools such as RegulomeDB [60] and HaploReg [61] have annotated all known SNPs with known functional elements in a variety of cell lineages and produce a score based on the likelihood that a particular SNP affects a functional element.

Nevertheless, identifying the targets of enhancers has been challenging. Although it was established very early on that enhancers regulate genes at a distance, it wasn’t immediately clear how this activity was mediated. With the development of chromatin conformation techniques, it was demonstrated that enhancers interact physically with their target promoters and that these interactions were cell-type specific. A summary of chromatin conformation techniques is presented in Table 3. With the development of more advanced techniques and higher resolution Hi-C data, it was shown that the contact domains and loops are highly variable and cell-type specific [68–70]. Other studies have shown that it is possible to reconstruct the lineage of primary blood cell types by interactions alone, and that these interactions are highly cell-type specific [23] and change with differentiation [71, 72] and activation [73]. Moreover, they found that the number or intensity of interactions with active enhancers correlated with expression levels of genes [23, 73, 74]. We now know that multiple enhancers can interact simultaneously with a single promoter [75] and that a single enhancer can regulate multiple genes at the same time within the same chromatin domain [76]. Live imaging studies have also shown that interactions are highly dynamic and transient [76, 77]. Recent evidence gathered using ChIP-Seq and RNA-Seq data from a large set of genotyped cell-lines has shown that regulatory activity is highly associated within well-delimited cis-regulatory domains. These domains respect many features found in chromatin conformation, such as topologically associating domains (TADs), interaction intensity and compartments [78]. Moreover, they find that the activity and structure of these cis-regulatory domains are partly determined by the genetic makeup of individuals [78]. Similarly, a study has shown that genetic variants are associated with differences in chromatin conformation [79].

**Table 3:** Summary of the techniques used to analyse chromatin conformation

Technique	Description	Type
3C [62]	First technique developed on which future techniques were based. The chromatin is first digested with enzymes and then re-ligated such that interacting regions are re-ligated together. The resulting products are analysed by quantitative polymerase chain reaction (qPCR) to quantify the frequency of interactions	One to one
4C [63]	Same as 3C, but resulting products are analysed by microarray to test the interactions originating from one region with the rest of the genome.	One to all
Hi-C [64]	Same as 3C, but the resulting products are fragmented and sequenced. This produces the most comprehensive analysis genome wide, but requires significant sequencing efforts to map all possible interactions across the whole genome.	All to all
Capture Hi-C [65]	Same as Hi-C, but the library is first enriched for specific regions to focus the sequencing efforts to regions of interest such as promoters or disease-associated loci.	Many to all
ChIA-PET and HiChIP [26, 27, 66, 67]	Same as Hi-C, but the library is enriched using a chromatin immunoprecipitation step; for example, markers of active regions of the genome. HiChIP is similar to ChIA-PET but provides significant improvements over it.	Many to many/all

These studies have also begun to link disease-associated variants to specific interacting genes and have shown that it is possible to use this information to prioritize genetic targets from non-coding GWAS SNPs. Importantly, this list of variant-gene interactions rediscovers 25% of previously reported eQTLs [23]. Because the 3 D chromatin conformation and enhancer-promoter interactome is highly context specific, with many enhancers regulating different genes in different tissues, many studies have interrogated the enhancer-promoter interactome on disease specific tissues such as cardio-myocytes [80, 81] for cardiovascular diseases and pancreatic islets [82, 83] for diabetes, and have shown that disease-associated loci are significantly enriched in regulatory regions active in those tissues. In their recent publication, Montefiori *et al.* linked 1999 cardiovascular disease-associated SNPs to 347 target genes in human-induced pluripotent stem cell derived cardio-myocytes, remarkably showing that 90% of variants did not target the nearest gene [80]. More recently, a group has shown that genetic variants associated with Type 1 Diabetes alter the chromatin conformation at disease-associated loci in a mouse model [84].

Other studies used region CHi-C [24, 25, 28, 65, 85, 86], targeting specific disease-associated loci to better identify causal genes that interacted with non-coding regulatory elements that could be disrupted by the variants. To date, only one study has investigated PsA-associated loci [24]; in two cell lines (B and T cells) 116 regions associated with autoimmune diseases including PsA were found to interact with at least one gene promoter. For example, the locus 6q23, which contains variants linked to PsA, RA, SLE, celiac disease, T1D, IBD and Ps, was previously assigned to *TNFAIP3*. In their work, McGovern *et al.* showed that this region also interacts with *IL20RA*, as well as showing a significant eQTL effect [25]. Another variant associated with PsA within the *DENND1B* gene was linked to *PTPRC,* a gene previously shown to be involved in RA [24]*.* A summary of the results from this paper for PsA-associated loci is available in Table 4. Although previous studies have shown strong evidence for the use of disease-relevant tissues, no study to date has used tissues derived from PsA patients.

**Table 4:** Genes linked to PsA-associated loci via region capture Hi-C

Lead SNP	Interacting genes
rs2020854	*COQ10A, BAZ2A, ANKRD52, NABP2, ORMDL2, ZC3H10, SMARCC2, RP11, SLC39A5, CS, RNF41, PAN2, PA2G4, RN7SL770P, ESYT1, GLS2, MIP, TIMELESS, SPRYD4, SARNP*
rs33980500	*RP11, REV3L, KIAA1919, TUBE1*
rs4921482	*ADRA1B, GAPDHP40*
rs76956521	*ANXA6*

Data from CD4 T cells and B cells obtained from Martin *et al.* [24].

Experimental confirmation is required to confirm the effect of the putative enhancers on the identified genes. Currently available methods include eQTL analysis, measuring the effect that deletions of the enhancers can have on the expression of interacting target genes, and other gene editing (CRISPR) derived methods that use fusion proteins to specifically activate or repress enhancers [41, 87, 88]. In a recent study, Mumbach *et al.* used a deactivated Cas9 protein (dCas9) fused with a KRAB domain that functions as a repressor (CRISPRi) to target three enhancers and show that it caused a reduction of transcription from interacting genes [27].

Another limitation that is often present in chromatin conformation studies is the separation of functional annotation and chromatin conformation analysis. Most use publicly available annotation [58, 59] to annotate results and the majority use either cell lines or primary cells from healthy donors instead of patient samples. This can lead to missing important regulatory elements that could be disease specific.

Nevertheless, combining different forms of functional genomics studies has the potential to translate the results from GWAS studies into understanding of the mechanisms of disease (Fig. 1).

## Impact on drug discovery

Treatment options for PsA and other autoimmune diseases are limited and often not effective for all patients. In particular, most current treatment options are composed of broad-spectrum anti-inflammatory drugs or target very few pathways (Table 5). This is a result of the poor understanding of the mechanisms and pathways involved in the disease and the high failure rate in drug development. Right now, only 10% of drugs that start clinical trials reach patients, with >50% failing at late stages [90, 91], primarily due to inadequate efficacy [90]. This has led to extremely high cost of drug development and stagnation of new development due to the high economic risk.

**Table 5:** Currently available drugs for PsA [89]

Drug	Mechanism
Nonsteroidal anti-inflammatory drugs (NSAIDs)	Anti-inflammatory
Corticosteroids	Anti-cortisol / Anti-inflammatory
Methotrexate	Inhibition of purine metabolism – inhibition of T-cell activation
Sulfasalazine	Immunosuppressive
Cyclosporine	T-cell suppressant / calcineurin–phosphatase pathway inhibition
Leflunomide	pyrimidine synthesis inhibitor / slows rapidly replicating lymphocytes
Etanercept, adalimumab, golimumab, infliximab and certolizumab	TNF alpha inhibitors
Abatacept	B7 protein inhibitor(antagonist) / blocks activation of T-cells
Ustekinumab	IL-12 and IL-23 inhibitor

Recently, a new wave of optimism has been brought thanks to the genetic dissection of complex diseases. Contrary to traditional methods of target identification, which use phenotypic data that are subject to confounding and environmental effects, genetic susceptibility factors are stable biomarkers that can provide clues to causality as well as providing information about pathways that are perturbed in disease and therefore could be targets for therapy. Moreover, GWAS studies have been designed from the ground up to obtain high statistical confidence thanks to factors such as adequate correction for multiple testing and appropriate sample sizes. This resulted in high reproducibility of results [92], which is in contrast to the current medical research trend [93, 94].

About 22% of the protein-coding genes are druggable by conventional drugs [95], and the percentage could become even higher as methods based on RNA inhibition are developed. Moreover, repurposing of available drugs can significantly speed the path to patient benefit because they have already been safety tested and chemically characterized.

Different studies have begun to exploit GWAS results to produce a new list of possible drug targets for coronary artery disease (CAD) [96], Parkinson’s disease [97] and RA [98], often rediscovering most of the drugs currently in use for treating these disorders. Promising results have been already obtained with a number of diseases. In PsA, for example, the identification of genetic associations in the IL-23 pathway provided genetic evidence for the repositioning of biologic drugs targeting components of this pathway in PsA [99]. Another inhibitor of IL-17A originally developed for Ps, RA and uveitis has been repurposed for use in ankylosing spondylitis [100].

Most studies to date have not used functional genomics to link variants to candidate genes, relying often on empirical methods such as choosing the closest or overlapping genes. As explained previously, this can often lead to incorrect conclusions about the functional gene and, more frequently, missing out genes that could have been potentially targeted. This is starting to change with the development of new methods that utilize functional annotation and chromatin conformation to discover genes and pathways that can be targeted disease. A recent publication from Martin *et al.* has used CHi-C data to identify potential drug targets in RA, JIA and PsA [101]. Using their approach, they have rediscovered 48 known drug targets and 87 potential new drugs for PsA. Another recent publication has developed a new way to prioritize target genes using a network connectivity metric [102] and, by analysing genetic and functional data from 30 other immune traits, rediscovered many known targets and predicted activity in high-throughput screens.

## Conclusion

Over the last decade, genetic studies have identified a significant number of loci associated with PsA that have greatly improved our understanding of the disease mechanism. However, PsA has a low prevalence and is clinically heterogeneous and difficult to distinguish from psoriasis. For this reason, it has been understudied compared with other diseases such as RA, T2D and IBD for which there have been a great number of new loci identified and many more potential drug targets identified.

Moreover, there are still many obstacles to overcome to accomplish the full potential of functional genomics, such as limitation in current techniques and analysis methods, but recent discoveries, especially in the field of basic biology (such as chromatin conformation regulation), will surely provide a new wave of functional targets (or discoveries) for complex diseases.

Thanks to these we will have a better understanding of the underlying mechanism of the diseases, the cell types actively involved in disease progression and discover novel drug targets that can expand our repertoire of tools to combat PsA. A better understanding of the disease and its genetics will also aid in the stratification of patients for existing therapies, which is going to be particularly important in a disease that is as varied as PsA.

## References

[1] Coulton BL, Thomson K, Symmons DPM, Popert AJ. Outcome in patients hospitalised for psoriatic arthritis. Clin Rheumatol 1989;8:261–5. 2758772 10.1007/BF02030083

[2] Zink A, Thiele K, Huscher D et al. Healthcare and burden of disease in psoriatic arthritis. A comparison with rheumatoid arthritis and ankylosing spondylitis. J Rheumatol 2006;33:86–90. 16395755

[3] Huscher D, Merkesdal S, Thiele K et al. Cost of illness in rheumatoid arthritis, ankylosing spondylitis, psoriatic arthritis and systemic lupus erythematosus in Germany. Ann Rheum Dis 2006;65:1175–83. 16540552 10.1136/ard.2005.046367 PMC1798296

[4] Husted JA, Gladman DD, Farewell VT, Cook RJ. Health-related quality of life of patients with psoriatic arthritis: a comparison with patients with rheumatoid arthritis. Arthritis Rheum 2001;45:151–8. 11324779 10.1002/1529-0131(200104)45:2<151::AID-ANR168>3.0.CO;2-T

[5] Leung YY, Ogdie A, Orbai A-M et al. Classification and outcome measures for psoriatic arthritis. Front Med 2018;5:246. 10.3389/fmed.2018.00246 PMC6135872 30238006

[6] Wong PCH, Leung Y-Y, Li EK, Tam L-S. Measuring disease activity in psoriatic arthritis. Int J Rheumatol 2012;2012:839425. 23319952 10.1155/2012/839425 PMC3540792

[7] O'Rielly DD, Rahman P. Genetics of susceptibility and treatment response in psoriatic arthritis. Nat Rev Rheumatol 2011;7:718–32. 22064628 10.1038/nrrheum.2011.169

[8] Veale DJ, Fearon U. The pathogenesis of psoriatic arthritis. Lancet 2018;391:2273–84. 29893226 10.1016/S0140-6736(18)30830-4

[9] Rahman P. Genetic epidemiology of psoriasis and psoriatic arthritis. Ann Rheum Dis 2005;64:ii37–9. 15708933 10.1136/ard.2004.030775 PMC1766868

[10] Moll JM, Wright V. Familial occurrence of psoriatic arthritis. Ann Rheum Dis 1973;32:181–201. 4715537 10.1136/ard.32.3.181 PMC1006078

[11] Chandran V Schentag CT Brockbank JE et al. Familial aggregation of psoriatic arthritis. Ann Rheum Dis 2009;68:664–7. 18524791 10.1136/ard.2008.089367

[12] Myers A, Kay LJ, Lynch SA, Walker DJ. Recurrence risk for psoriasis and psoriatic arthritis within sibships. Rheumatology 2005;44:773–6. 15757963 10.1093/rheumatology/keh589

[13] Karason A, Love TJ, Gudbjornsson B. A strong heritability of psoriatic arthritis over four generations–the Reykjavik Psoriatic Arthritis Study. Rheumatology 2009;48:1424–8. 19741010 10.1093/rheumatology/kep243

[14] Stuart PE, Nair RP, Tsoi LC et al. Genome-wide association analysis of psoriatic arthritis and cutaneous psoriasis reveals differences in their genetic architecture. Am J Hum Genet 2015;97:816–36. 26626624 10.1016/j.ajhg.2015.10.019 PMC4678416

[15] Ellinghaus E, Stuart PE, Ellinghaus D et al. Genome-wide meta-analysis of psoriatic arthritis identifies susceptibility locus at REL. J Invest Dermatol 2012;132:1133–40. 22170493 10.1038/jid.2011.415 PMC3305829

[16] Liu Y, Helms C, Liao W et al. A genome-wide association study of psoriasis and psoriatic arthritis identifies new disease loci. PLoS Genet 2008;4:e1000041. 18369459 10.1371/journal.pgen.1000041 PMC2274885

[17] Ellinghaus E, Ellinghaus D, Stuart PE et al. Genome-wide association study identifies a psoriasis susceptibility locus at TRAF3IP2. Nat Genet 2010;42:991–5. 20953188 10.1038/ng.689 PMC3136364

[18] Nair RP, fDuffin KC, Helms C et al. Genome-wide scan reveals association of psoriasis with IL-23 and NF-κB pathways. Nat Genet 2009;41:199–204. 19169254 10.1038/ng.311 PMC2745122

[19] Bowes J, Budu-Aggrey A, Huffmeier U et al. Dense genotyping of immune-related susceptibility loci reveals new insights into the genetics of psoriatic arthritis. Nat Commun 2015;6:6046. 25651891 10.1038/ncomms7046 PMC4327416

[20] Bowes J, Loehr S, Budu-Aggrey A et al. PTPN22 is associated with susceptibility to psoriatic arthritis but not psoriasis: evidence for a further PsA-specific risk locus. Ann Rheum Dis 2015;74:1882–5. 25923216 10.1136/annrheumdis-2014-207187 PMC4602265

[21] Winchester R, Minevich G, Steshenko V et al. HLA associations reveal genetic heterogeneity in psoriatic arthritis and in the psoriasis phenotype. Arthritis Rheum 2012;64:1134–44. 22006066 10.1002/art.33415

[22] Farh KK-H, Marson A, Zhu J et al. Genetic and epigenetic fine mapping of causal autoimmune disease variants. Nature 2015;518:337–43. 25363779 10.1038/nature13835 PMC4336207

[23] Javierre BM, Burren OS, Wilder SP et al. Lineage-specific genome architecture links enhancers and non-coding disease variants to target gene promoters. Cell 2016;167:1369–84.e19. 27863249 10.1016/j.cell.2016.09.037 PMC5123897

[24] Martin P, McGovern A, Orozco G et al. Capture Hi-C reveals novel candidate genes and complex long-range interactions with related autoimmune risk loci. Nat Commun 2015;6:7. 10.1038/ncomms10069 PMC4674669 26616563

[25] McGovern A, Schoenfelder S, Martin P et al. Capture Hi-C identifies a novel causal gene, *IL20RA*, in the pan-autoimmune genetic susceptibility region 6q23. Genome Biol 2016;17:212. 27799070 10.1186/s13059-016-1078-x PMC5088679

[26] Mumbach MR, Rubin AJ, Flynn RA et al. HiChIP: efficient and sensitive analysis of protein-directed genome architecture. Nat Methods 2016;13:919–22. 27643841 10.1038/nmeth.3999 PMC5501173

[27] Mumbach MR, Satpathy AT, Boyle EA et al. Enhancer connectome in primary human cells identifies target genes of disease-associated DNA elements. Nat Genet 2017;49:1602–12. 28945252 10.1038/ng.3963 PMC5805393

[28] Martin P, McGovern A, Massey J et al. Identifying causal genes at the multiple sclerosis associated region 6q23 using capture Hi-C. PLoS One 2016;11:e0166923. 27861577 10.1371/journal.pone.0166923 PMC5115837

[29] Nolis IK, McKay DJ, Mantouvalou E et al. Transcription factors mediate long-range enhancer-promoter interactions. Proc Natl Acad Sci USA 2009;106:20222–7. 19923429 10.1073/pnas.0902454106 PMC2779200

[30] Chen H, Levo M, Barinov L et al. Dynamic interplay between enhancer–promoter topology and gene activity. Nat Genet 2018;50:1296–303. 30038397 10.1038/s41588-018-0175-z PMC6119122

[31] Bogunia-Kubik K, Wysoczańska B, Piątek D et al. Significance of polymorphism and expression of miR-146a and NFkB1 genetic variants in patients with rheumatoid arthritis. Arch Immunol Ther Exp 2016;64:131–6. 10.1007/s00005-016-0443-5 PMC5334424 28083614

[32] Messemaker TC, Frank-Bertoncelj M, Marques RB et al. A novel long non-coding RNA in the rheumatoid arthritis risk locus TRAF1-C5 influences C5 mRNA levels. Genes Immun 2016;17:85–92. 26673966 10.1038/gene.2015.54

[33] GTEx Consortium. The Genotype-Tissue Expression (GTEx) project. Nat Genet 2013;45:580–5. 23715323 10.1038/ng.2653 PMC4010069

[34] Joehanes R, Zhang X, Huan T et al. Integrated genome-wide analysis of expression quantitative trait loci aids interpretation of genomic association studies. Genome Biol 2017;18:16. 28122634 10.1186/s13059-016-1142-6 PMC5264466

[35] Võsa U, Claringbould A, Westra H-J et al. Unraveling the polygenic architecture of complex traits using blood eQTL metaanalysis. bioRxiv. 2018; 447367.

[36] Westra H-J, Peters MJ, Esko T et al. Systematic identification of trans eQTLs as putative drivers of known disease associations. Nat Genet 2013;45:1238–43. 24013639 10.1038/ng.2756 PMC3991562

[37] De Jager PL, Hacohen N, Mathis D et al. ImmVar project: insights and design considerations for future studies of “healthy” immune variation. Semin Immunol 2015;27:51–7. 25819567 10.1016/j.smim.2015.03.003

[38] Schmiedel BJ, Singh D, Madrigal A et al. Impact of genetic polymorphisms on human immune cell gene expression. Cell 2018;175:1701–15.e16. 30449622 10.1016/j.cell.2018.10.022 PMC6289654

[39] Lappalainen T, Sammeth M, Friedländer MR et al. Transcriptome and genome sequencing uncovers functional variation in humans. Nature 2013;501:506–11. 24037378 10.1038/nature12531 PMC3918453

[40] Alasoo K, Rodrigues J, Mukhopadhyay S et al. Shared genetic effects on chromatin and gene expression indicate a role for enhancer priming in immune response. Nat Genet 2018;50:424–31. 29379200 10.1038/s41588-018-0046-7 PMC6548559

[41] Simeonov DR, Gowen BG, Boontanrart M et al. Discovery of stimulation-responsive immune enhancers with CRISPR activation. Nature 2017;549:111–5. 28854172 10.1038/nature23875 PMC5675716

[42] Nica AC, Dermitzakis ET. Expression quantitative trait loci: present and future. Philos Trans R Soc Lond B Biol Sci 2013;368:20120362. 23650636 10.1098/rstb.2012.0362 PMC3682727

[43] Battle A, Brown CD, Engelhardt BE et al. Genetic effects on gene expression across human tissues. Nature 2017;550:204–13. 29022597 10.1038/nature24277 PMC5776756

[44] Fairfax BP, Humburg P, Makino S et al. Innate immune activity conditions the effect of regulatory variants upon monocyte gene expression. Science 2014;343:1246949. 24604202 10.1126/science.1246949 PMC4064786

[45] Thalayasingam N, Nair N, Skelton AJ et al. CD4+ and B lymphocyte expression quantitative traits at rheumatoid arthritis risk loci in patients with untreated early arthritis. Arthritis Rheumatol 2018;70:361–70. 29193869 10.1002/art.40393 PMC5888199

[46] Ding J, Gudjonsson JE, Liang L et al. Gene expression in skin and lymphoblastoid cells: refined statistical method reveals extensive overlap in cis-eQTL signals. Am J Hum Genet 2010;87:779–89. 21129726 10.1016/j.ajhg.2010.10.024 PMC2997368

[47] Kasela S, Kisand K, Tserel L et al. Pathogenic implications for autoimmune mechanisms derived by comparative eQTL analysis of CD4+ versus CD8+ T cells. PLoS Genet 2017;13:e1006643. 28248954 10.1371/journal.pgen.1006643 PMC5352142

[48] Nicolae DL, Gamazon E, Zhang W et al. Trait-associated SNPs are more likely to be eQTLs: annotation to enhance discovery from GWAS. PLoS Genet 2010;6:e1000888. 20369019 10.1371/journal.pgen.1000888 PMC2848547

[49] Pelikan RC, Kelly JA, Fu Y et al. Enhancer histone-QTLs are enriched on autoimmune risk haplotypes and influence gene expression within chromatin networks. Nat Commun 2018;9:2905. 30046115 10.1038/s41467-018-05328-9 PMC6060153

[50] McVicker G, van de Geijn B, Degner JF et al. Identification of genetic variants that affect histone modifications in human cells. Science 2013;342:747–9. 24136359 10.1126/science.1242429 PMC3947669

[51] Kumasaka N, Knights AJ, Gaffney DJ. High-resolution genetic mapping of putative causal interactions between regions of open chromatin. Nat Genet 2019;51:128–137. 30478436 10.1038/s41588-018-0278-6 PMC6330062

[52] Gate RE, Cheng CS, Aiden AP et al. Genetic determinants of co-accessible chromatin regions in activated T cells across humans. Nat Genet 2018;50:1140–1150. 29988122 10.1038/s41588-018-0156-2 PMC6097927

[53] Calderon D, Nguyen MLT, Mezger A et al. Landscape of stimulation-responsive chromatin across diverse human immune cells. Nat Genet 2019;51:1494–1505. 10.1038/s41588-019-0505-9 PMC6858557 31570894

[54] Yao L, Berman BP, Farnham PJ. Demystifying the secret mission of enhancers: linking distal regulatory elements to target genes. Crit Rev Biochem Mol Biol 2015;50:550–73. 26446758 10.3109/10409238.2015.1087961 PMC4666684

[55] Shlyueva D, Stampfel G, Stark A. Transcriptional enhancers: from properties to genome-wide predictions. Nat Rev Genet 2014;15:272–86. 24614317 10.1038/nrg3682

[56] Bulger M, Groudine M. Functional and mechanistic diversity of distal transcription enhancers. Cell 2011;144:327–39. 21295696 10.1016/j.cell.2011.01.024 PMC3742076

[57] Banerji J, Rusconi S, Schaffner W. Expression of a β-globin gene is enhanced by remote SV40 DNA sequences. Cell 1981;27:299–308. 6277502 10.1016/0092-8674(81)90413-x

[58] Kundaje A, Meuleman W, Ernst J et al. Integrative analysis of 111 reference human epigenomes. Nature 2015;518:317–30. 25693563 10.1038/nature14248 PMC4530010

[59] The ENCODE Project Consortium. An integrated encyclopedia of DNA elements in the human genome. Nature 2012;489:57–74. 22955616 10.1038/nature11247 PMC3439153

[60] Boyle AP, Hong EL, Hariharan M et al. Annotation of functional variation in personal genomes using RegulomeDB. Genome Res 2012;22:1790–7. 22955989 10.1101/gr.137323.112 PMC3431494

[61] Ward LD, Kellis M. HaploReg: a resource for exploring chromatin states, conservation, and regulatory motif alterations within sets of genetically linked variants. Nucleic Acids Res 2012;40:D930–4. 22064851 10.1093/nar/gkr917 PMC3245002

[62] Dekker J, Rippe K, Dekker M, Kleckner N. Capturing chromosome conformation. Science 2002;295:1306–11. 11847345 10.1126/science.1067799

[63] Simonis M, Klous P, Splinter E et al. Nuclear organization of active and inactive chromatin domains uncovered by chromosome conformation capture-on-chip (4C). Nat Genet 2006;38:1348–54. 17033623 10.1038/ng1896

[64] Lieberman-Aiden E, van Berkum NL, Williams L et al. Comprehensive mapping of long-range interactions reveals folding principles of the human genome. Science 2009;326:289–93. 19815776 10.1126/science.1181369 PMC2858594

[65] Dryden NH, Broome LR, Dudbridge F et al. Unbiased analysis of potential targets of breast cancer susceptibility loci by Capture Hi-C. Genome Res 2014;24:1854–68. 25122612 10.1101/gr.175034.114 PMC4216926

[66] Fullwood MJ, Liu MH, Pan YF et al. An oestrogen-receptor-α-bound human chromatin interactome. Nature 2009;462:58–64. 19890323 10.1038/nature08497 PMC2774924

[67] Fullwood MJ, Han Y, Wei CL, Ruan X, Ruan Y. Chromatin interaction analysis using paired-end tag sequencing. Curr Protoc Mol Biol 2010;21:21.15.1–25. 10.1002/0471142727.mb2115s89 PMC6924956 20069536

[68] Rao SSP, Huntley MH, Durand NC et al. A 3D map of the human genome at kilobase resolution reveals principles of chromatin looping. Cell 2014;159:1665–80. 25497547 10.1016/j.cell.2014.11.021 PMC5635824

[69] Schmitt AD, Hu M, Jung I et al. A compendium of chromatin contact maps reveals spatially active regions in the human genome. Cell Rep 2016;17:2042–59. 27851967 10.1016/j.celrep.2016.10.061 PMC5478386

[70] Hansen AS, Cattoglio C, Darzacq X, Tjian R. Recent evidence that TADs and chromatin loops are dynamic structures. Nucleus 2018;9:20–32. 29077530 10.1080/19491034.2017.1389365 PMC5990973

[71] Siersbæk R, Madsen JGS, Javierre BM et al. Dynamic rewiring of promoter-anchored chromatin loops during adipocyte differentiation. Mol Cell 2017;66:420–435.e5. 28475875 10.1016/j.molcel.2017.04.010

[72] Rubin AJ, Barajas BC, Furlan-Magaril M et al. Lineage-specific dynamic and pre-established enhancer–promoter contacts cooperate in terminal differentiation. Nat Genet 2017;49:1522–8. 28805829 10.1038/ng.3935 PMC5715812

[73] Burren OS, Rubio García A, Javierre B-M et al. Chromosome contacts in activated T cells identify autoimmune disease candidate genes. Genome Biol 2017;18:165. 28870212 10.1186/s13059-017-1285-0 PMC5584004

[74] Greenwald WW, Li H, Benaglio P et al. Subtle changes in chromatin loop contact propensity are associated with differential gene regulation and expression. Nat Commun 2019;10:1054. 30837461 10.1038/s41467-019-08940-5 PMC6401380

[75] Oudelaar AM, Davies JOJ, Hanssen LLP et al. Single-allele chromatin interactions identify regulatory hubs in dynamic compartmentalized domains. Nat Genet 2018;50:1744–1751. 30374068 10.1038/s41588-018-0253-2 PMC6265079

[76] Fukaya T, Lim B, Levine M. Enhancer control of transcriptional bursting. Cell 2016;166:358–68. 27293191 10.1016/j.cell.2016.05.025 PMC4970759

[77] Bartman CR, Hsu SC, Hsiung CC-S, Raj A, Blobel GA. Enhancer regulation of transcriptional bursting parameters revealed by forced chromatin looping. Mol Cell 2016;62:237–47. 27067601 10.1016/j.molcel.2016.03.007 PMC4842148

[78] Delaneau O, Zazhytska M, Borel C et al. Chromatin three-dimensional interactions mediate genetic effects on gene expression. Science 2019;364:eaat8266. 31048460 10.1126/science.aat8266

[79] Gorkin DU, Qiu Y, Hu M et al. Common DNA sequence variation influences 3-dimensional conformation of the human genome. Genome Biol 2019;20:255. 31779666 10.1186/s13059-019-1855-4 PMC6883528

[80] Montefiori LE, Sobreira DR, Sakabe NJ et al. A promoter interaction map for cardiovascular disease genetics. Elife 2018;7:e35788. 29988018 10.7554/eLife.35788 PMC6053306

[81] Choy M-K, Javierre BM, Williams SG et al. Promoter interactome of human embryonic stem cell-derived cardiomyocytes connects GWAS regions to cardiac gene networks. Nat Commun 2018;9:2526. 29955040 10.1038/s41467-018-04931-0 PMC6023870

[82] Miguel-Escalada I, Bonàs-Guarch S, Cebola I et al. Human pancreatic islet three-dimensional chromatin architecture provides insights into the genetics of type 2 diabetes. Nat Genet 2019;51:1137–48. 31253982 10.1038/s41588-019-0457-0 PMC6640048

[83] Greenwald WW, Chiou J, Yan J et al. Pancreatic islet chromatin accessibility and conformation reveals distal enhancer networks of type 2 diabetes risk. Nat Commun 2019;10:2078. 31064983 10.1038/s41467-019-09975-4 PMC6505525

[84] Fasolino M, Goldman N, Wang W et al. Genetic variation in type 1 diabetes reconfigures the 3D chromatin organization of T cells and alters gene expression. Immunity 2020;52:257–274.e11. 32049053 10.1016/j.immuni.2020.01.003 PMC7152927

[85] Jäger R, Migliorini G, Henrion M et al. Capture Hi-C identifies the chromatin interactome of colorectal cancer risk loci. Nat Commun 2015;6:6178. 25695508 10.1038/ncomms7178 PMC4346635

[86] Cairns J, Freire-Pritchett P, Wingett SW et al. CHiCAGO: robust detection of DNA looping interactions in Capture Hi-C data. Genome Biol 2016;17:127. 27306882 10.1186/s13059-016-0992-2 PMC4908757

[87] Hilton IB, D'Ippolito AM, Vockley CM et al. Epigenome editing by a CRISPR-Cas9-based acetyltransferase activates genes from promoters and enhancers. Nat Biotechnol 2015;33:510–7. 25849900 10.1038/nbt.3199 PMC4430400

[88] Gilbert LA, Larson MH, Morsut L et al. CRISPR-mediated modular RNA-guided regulation of transcription in eukaryotes. Cell 2013;154:442–51. 23849981 10.1016/j.cell.2013.06.044 PMC3770145

[89] Psoriatic Arthritis Treatment [Internet]. https://www.arthritis.org/about-arthritis/types/psoriatic-arthritis/treatment.php (30 November 2018, date last accessed).

[90] Hwang TJ, Carpenter D, Lauffenburger JC et al. Failure of investigational drugs in late-stage clinical development and publication of trial results. JAMA Intern Med 2016;176:1826. 27723879 10.1001/jamainternmed.2016.6008

[91] Plenge RM, Scolnick EM, Altshuler D. Validating therapeutic targets through human genetics. Nat Rev Drug Discov 2013;12:581–94. 23868113 10.1038/nrd4051

[92] Marigorta UM, Rodríguez JA, Gibson G, Navarro A. Replicability and prediction: lessons and challenges from GWAS. Trends Genet 2018;34:504–17. 29716745 10.1016/j.tig.2018.03.005 PMC6003860

[93] Ioannidis J. Why most published research findings are false. PLoS Med 2005;2:e124. 16060722 10.1371/journal.pmed.0020124 PMC1182327

[94] Flier JS. Irreproducibility of published bioscience research: diagnosis, pathogenesis and therapy. Mol Metab 2017;6:2–9. 28123930 10.1016/j.molmet.2016.11.006 PMC5220388

[95] Finan C, Gaulton A, Kruger FA et al. The druggable genome and support for target identification and validation in drug development. Sci Transl Med 2017;9:eaag1166. 28356508 10.1126/scitranslmed.aag1166 PMC6321762

[96] Shu L, Blencowe M, Yang X. Translating GWAS findings to novel therapeutic targets for coronary artery disease. Front Cardiovasc Med 2018;5:56. 29900175 10.3389/fcvm.2018.00056 PMC5989327

[97] Uenaka T, Satake W, Cha P-C et al. In silico drug screening by using genome-wide association study data repurposed dabrafenib, an anti-melanoma drug, for Parkinson’s disease. Hum Mol Genet 2018;27:3974–85. 30137437 10.1093/hmg/ddy279 PMC6216208

[98] Okada Y, Wu D, Trynka G et al. Genetics of rheumatoid arthritis contributes to biology and drug discovery. Nature 2014;506:376–81. 24390342 10.1038/nature12873 PMC3944098

[99] McInnes IB, Sieper J, Braun J et al. Efficacy and safety of secukinumab, a fully human anti-interleukin-17A monoclonal antibody, in patients with moderate-to-severe psoriatic arthritis: a 24-week, randomised, double-blind, placebo-controlled, phase ii proof-of-concept trial. Ann Rheum Dis 2014;73:349–56. 23361084 10.1136/annrheumdis-2012-202646

[100] Jethwa H, Bowness P. The interleukin (IL)-23/IL-17 axis in ankylosing spondylitis: new advances and potentials for treatment. Clin Exp Immunol 2016;183:30–6. 26080615 10.1111/cei.12670 PMC4687521

[101] Martin P, Ding J, Duffus K et al. Chromatin interactions reveal novel gene targets for drug repositioning in rheumatic diseases. Ann Rheum Dis 2019;78:1127–34. 31092410 10.1136/annrheumdis-2018-214649 PMC6691931

[102] Fang H, De Wolf H, Knezevic B et al. A genetics-led approach defines the drug target landscape of 30 immune-related traits. Nat Genet 2019;51:1082–1091. 31253980 10.1038/s41588-019-0456-1 PMC7124888

